# Demonstrating the utility of *Escherichia coli* asymptomatic bacteriuria isolates’ virulence profile towards diagnosis and management—A preliminary analysis

**DOI:** 10.1371/journal.pone.0267296

**Published:** 2022-05-06

**Authors:** Lalitha Maniam, Kumutha Malar Vellasamy, Hassan Mahmood Jindal, Vallikannu Narayanan, Mahmoud Danaee, Jamuna Vadivelu, Vinod Pallath

**Affiliations:** 1 Department of Medical Microbiology, Faculty of Medicine, University of Malaya, Kuala Lumpur, Malaysia; 2 Department of Obstetrics and Gynaecology, Faculty of Medicine, University of Malaya, Kuala Lumpur, Malaysia; 3 Department of Social and Preventive Medicine, Faculty of Medicine, University of Malaya, Kuala Lumpur, Malaysia; 4 Medical Education Research and Development Unit (MERDU), Faculty of Medicine, University of Malaya, Kuala Lumpur, Malaysia; Suez Canal University, EGYPT

## Abstract

Asymptomatic bacteriuria (ASB) caused by *Escherichia coli (E*. *coli)* is a significant condition associated with pregnancy and is considered as prognostic for the development of symptomatic urinary tract infection (UTI). However, treating all ASB increases the use of antibiotics and leads to the development of multidrug resistance (MDR). Therefore, this study aimed to identify the distribution of UPEC associated virulence genes and antibiotic susceptibility among phylogroups of *E*. *coli* isolated from ASB in pregnancy. Moreover, the gene expression of selected virulence genes was also compared among two *E*. *coli* isolates (with different pathogenic potential) to determine its pathogenicity. One hundred and sixty *E*. *coli* isolates from midstream urine samples of pregnant women with ASB were subjected to PCR-based detection for its phylogroups and virulence genes. The antibiotic susceptibility of isolated strains was determined by the disc diffusion method. Expression of the virulence genes were determined through microarray analysis and quantitative Real-Time PCR. The prevalence of ASB in this study was 16.1%. Within ASB isolates, the occurrence of phylogroup B2 was the highest, and isolates from this group harboured most of the virulence genes studied. Overall, the most identified virulence genes among all phylogroups in descending order were *fimH*, *chuA*, *kpsMTII*, *usp*, *fyuA*, *hlyA*, *iroN*, *cnf*, *papC*, *sfa*, *ompT*, and *sat*. In this study, higher resistance to antibiotics was observed for ampicillin (77.5%), amoxicillin-clavulanate (54.4%), trimethoprim-sulfamethoxazole (46.9%) and amikacin (43.8%) compared to the other tested antibiotics and 51.9% of the tested isolates were MDR. Furthermore, hierarchical clustering and gene expression analysis demonstrated extreme polarization of pathogenic potential of *E*. *coli* causing ASB in pregnancy necessitating the need for bacterial isolate focused approach towards treatment of ASB.

## Introduction

Asymptomatic bacteriuria (ASB) is defined as the excretion of a significant number (≥ 10^5^ CFU/mL) of bacteria in midstream urine, in the absence of signs and symptoms of UTI. It is broadly accepted as a benign condition in healthy individuals, thus will be not be screened and treated [1]. However, ASB is considered as a significant associated condition in pregnancy due to its potential risk factor for the development of symptomatic UTI and its adverse consequences [2–4]. The incidence of ASB during pregnancy has been reported to be approximately 2–11% [5]. Pregnant women are more vulnerable to getting ASB mainly due to the changes that happen during pregnancy such as the mechanical and anatomical changes (compression from the enlarging uterus on the bladder), hormonal changes (increase in progesterone and estrogen), hydroureter, and hydronephrosis [5]. Besides, differences in urine pH and osmolarity, development of gestational diabetes, and aminoaciduria were also reported to further enhance the growth of bacteria in the urinary tract.

Screening and treatment for ASB in pregnancy have long been a part of the standard routine obstetric care to treat and clear the infection to reduce the risk of complications later during pregnancy [2, 6–8]. However, recently there were discrepancies in opinion on the utility of treating ASB for the reduction in the risk of perinatal outcome [3, 6, 9]. Although several studies demonstrated the potential of ASB to develop into upper UTIs (pyelonephritis) and predispose to preterm birth, some other observational studies failed to prove this association of ASB with incidence of maternal complications. [10–13]. The results are inconsistent with the very low quality of evidence related to the harms and benefits of treating ASB [2, 14].

*Escherichia coli* is the predominant agent responsible for nearly 80% of all asymptomatic and symptomatic UTI cases [9, 15–17]. Although *E*. *coli has* a predominant commensalic association at gastrointestinal tract of humans, they also can become Extra-intestinal pathogenic *E*. *coli* (ExPEC) upon acquisition of various virulence genes into its pathogenicity islands (PAI) through horizontal gene transfer (HGT). Acquisition of the virulence genes predisposes *E*. *coli* to become pathogenic, thus causing clinical infections including UTIs. *E*. *coli* associated with UTIs are known as Uropathogenic *E*. *coli* (UPEC). Genomic analysis of the UPEC revealed most of the isolates harboured several virulence genes associated with adhesion (facilitate the adherence and colonisation), iron acquisition (allows to scavenge iron from the environment), toxin production (aids to invade into host tissue) and capsule synthesis (involved in biofilm formation and protects against host innate defence mechanism). Among various virulence genes studied to date *fimH*, *papC*, *sfa*, *hlyA*, *cnf*, *sat*, *kpsMTII*, *ompT* and *usp*, were evidenced to be associated with UPEC, a group of ExPEC. Phylogenetically *E*. *coli* from phylogroups B2 and D were reported as ExPEC, and isolates from phylogroups A and B1were reported as commensals [18]. *E*. *coli* isolated from both asymptomatic and symptomatic UTIs were identified mostly to be ExPEC.

ASB in pregnancy warrants significant use of antibiotics. However, emergence of MDR has become a major public health threat [19, 20], especially with grave consequences in the treatment and management of ASB and UTIs. Historically the prevalence of antimicrobial resistance and MDR among *E*. *coli* have been associated with nosocomial infections, however, currently it is shown to be disseminated in the community. Recent data revealed that *E*. *coli* has developed resistance to most clinically relevant antimicrobial classes recommended to treat UTIs such as penicillins, cephalosporins, and carbapenems. Between 2013 to 2016, the prevalence of extended-spectrum beta-lactamase (ESBL) producing urine isolated *E*. *coli* were reported to be increased from 10% to 67.3% [21]. *E*. *coli* are known to acquire resistance to antimicrobials through HGT of resistance genes into its R-plasmid [22]. Acquisition and dissemination of the antimicrobial resistance were identified to occur through various routes of infection such as from animal to human via food supply, direct contact with the animal, human to human transmission, and through environmental sources [23, 24]. In most circumstances infections caused by MDR *E*. *coli* often predisposes to poor treatment outcomes such as increased risk of treatment failure, morbidity, mortality, risk of hospitalization, health care associated cost as well as increased use of broad spectrum antibiotics.

The patients with ASB may have carried the same strain for months to years devoid of developing a disease response, leaving the commensal-like bacteria to successfully co-evolve with the host in a niche with other microbial competitors. Thus, screening and treating the ASB will be more reasonable and relevant among high-risk category pregnant women such as those with diabetes, history of UTI, sickle cell disease, polycystic kidney, congenital renal abnormalities, and grand multiparity [25]. Furthermore, due to the alarming situation of the emergence of multidrug resistance (MDR) among Gram-negative bacteria such as *E*. *coli*, the prescription of antibiotics to all at risk and the majority of non-risk category pregnant women might further complicate the situation. Overtreatment with antibiotics during pregnancy may result in number of undesirable effects such as alteration of foetal flora, delay in early gastrointestinal colonization, interfere with baby’s immune system development, cause cardiovascular defects and cleft-lip/cleft palate [26]. Moreover, treating the ASB may also eliminate low virulence strains that suppress the development of uropathogens, thus indirectly promoting the development of symptomatic UTIs.

Many studies have suggested a strong association between the presence of virulence genes and ExPEC phylogroups [27, 28]. The acquisition of the virulence encoding genes will aid the survival of UPEC in the urinary tract, facilitating the establishment of UTI in the host. Thus, the severity of symptomatic UTIs manifestations is often associated with the acquisition and expression of the virulence encoding genes. Therefore, identification of these UPEC associated virulence genes among ASB *E*. *coli* strains will not only aid to predict the possibility of developing symptomatic UTI among ASB cases, but also can be used as a predictive marker to decide the need for antibiotic treatment. Although the use of antibiotics in the treatment of ASB in pregnant women is recommended, it could not be fully justified without knowing the nature of the organism associated. This is important as there has been some evidence on the beneficial aspects of bacterial colonization in preventing symptomatic UTIs [29, 30]. All these lead to the need of determining the nature of the organism including the virulence characteristics before the commencement of therapy among ASB.

Information regarding phylogroups, virulence characteristics, and antibiotic susceptibility of *E*. *coli* causing ASB in pregnancy is still scarce. The unavailability of genotypic data coupled with disparity in treatment becomes a grave situation of inappropriate management practices, which could potentially lead to significant complications and morbidities in the later life of the affected patients. To the best of our knowledge, there is no local data available on the ASB caused by *E*. *coli* and its molecular virulence characteristics in the Malaysian population.

This study aimed to (1) determine the occurrence of virulence genes, antibiotic sensitivity, and its relationship with different phylogroups of *E*. *coli*, (2) analyse the distribution of virulence genes using hierarchical clustering (according to virulence functional groups) and (3) determine the gene expression profile of the selected virulence genes among the selected ASB isolates. These data may aid the development of a prognostic combination of phylogroups and virulence determinants which could predict the development of symptomatic UTIs. Such predictive prognostic marker combinations will help in the development of a more accurate treatment strategy for ASB and reduce the unwanted use of antibiotics.

## Materials and methods

### Institutional review board statement

The study was approved by the Medical Research Ethics Committee of the University of Malaya Medical Centre (UMMC) (MRECID. NO: 201765–5313).

### Specimen collection

A total of 1315 specimens were obtained from pregnant women at the Department of Obstetrics and Gynaecology, University Malaya Medical Centre (Kuala Lumpur, Malaysia) from July 2017 to May 2018.

#### Inclusion criteria

Healthy pregnant women from any age, parity, and gestational age without any symptoms of UTI attending the antenatal clinic at the study setting were invited to enrol in this study.

#### Exclusion criteria

Pregnant women with recent symptoms of UTI and those who were with a recent history of antibiotics or had antibiotic therapy at least two weeks before sampling was excluded from this study.

The urine samples were collected as clean-catch midstream urine (15–25 mL). Participants were informed to collect the urine in a sterile universal specimen container using aseptic collection techniques. Informed and written consent was obtained from all patients.

### Sample size determination

To determine the sample size for this study we used a power study, which is a very useful and frequently used method in medical research to prove sample size adequacy for a study [31, 32]. The prevalence of ABU in Malaysia is reported to be 1.9% [33]. To obtain an appropriate sample size from this population, we used the following formula.

$$n=\frac{{\left(Z_{1-\beta }+{Z_{\alpha /2}}_{}\right)}^{2}\left(p\left(1-p\right)\right)}{d^{2}}$$

Where, n = required sample size

Z_1-β_ = Z value at power 1-β (Minimum power 80% value = 0.84)

Zα_/2_ = Standard Normal value at confidence level at 100 (1-α) % (ideal value is 1.96 at 95% CI)

p = referred prevalence for the study

d = margin of error (ideal value is 0.05 for estimated prevalence in range of 20–80% and around 0.03 for less common or very common events (<20% or >80%).

Therefore, considering 80% power of test, 95% confidence interval, 3% marginal error, and 2% prevalence rate, the sample size was estimated to be 148.

### Bacterial isolation and identification

The collected urine samples were streaked semi-quantitatively (using a calibrated loop) onto MacConkey’s agar (Hi-Media, India) and Eosin Methylene Blue agar (EMB) (Hi-Media, India). The urine cultures were then incubated for 24 h at 37°C. All *E*. *coli* suspected colonies with significant counts of ≥ 10^5^ CFU/mL was selected for this study and confirmed its identification based on its phenotypic as well as biochemical characteristics. Biochemical identification of the isolates were tested for catalase, oxidase, urease, indole, Methyl red, Voges-Proskauer and citrate as previously described [34]. The isolates were stored in 30% glycerol at -70°C for further use.

### Genomic DNA extraction

The template DNA for PCR was extracted from each *E*. *coli* isolate using the distilled-water boiling method as described in the literature [35]. Briefly, a single colony of bacterial isolate growing on nutrient agar was suspended in 100 μl of deionized water and boiled in a water bath for 10 minutes. Following centrifugation at 10,000 × g for 5 minutes, the supernatant was collected and 1 μl of the supernatant containing the DNA was used as a template for PCR amplification.

### Confirmation of identification

The identified isolates were also confirmed through conventional PCR using a species-specific set of primers (Table 1) targeting 16S rRNA gene of *E*. *coli* [36].

**Table 1:** Primer used for the phylogenotyping and *E*. *coli* identification (16s rRNA).

PCR Reaction	Primer	Primer sequence	Amplicon size (bp)
Quadruplex	*chuA*.*F*	ATGGTACCGGACGAACCAAC	288
	*chuA*.*R*	TGCCGCCAGTACCAAAGACA	
	*yjA*.*F*	CAAACGTGAAGTGTCAGGAG	211
	*yjA*.*R*	AATGCGTTCCTCAACCTGTG	
	*TsPE4C2*.*F*	CACTATTCGTAAGGTCATCC	152
	*TsPE4C2*.*R*	AGTTTATCGCTGCGGGTCGC	
	*Ace*.*K*.*F*	AACGCTATTCGCCAGCTTGC	400
	*ArpA1*.*R*	TCTCCCCATACCGTACGCTA	
Group C	*trpAgpC*.*F*	AGTTTTATGCCCAGTGCGAG	219
	*trpAgpC*.*R*	TCTGCGCCGGTCACGCCCC	
Group E	*ArpAgpE*.*F*	GATTCCATCTTGTCAAAATATGCC	301
	*ArpAgpE*.*R*	GAAAAGAAAAAGAATTCCCAAGAG	
Internal control	*trpBA*.*F*	CGGCGATAAAGACATCTTCAC	489
	*trpBA*.*R*	GCAACGCGGCCTGGCGGAAG	
16s rRNA	*16s rRNA*.*F*	CATGCCGCGTGTATGAAGAA	100
	*16s rRNA*.*R*	CGGGTAACGTCAATGAGCAAA	

### Phylogrouping

The allocation of *E*. *coli* isolates into phylogroups A, B1, B2, C, D, E, F, and clade 1 were performed according to the quadruplex PCR method using Clermont’s protocol [18]. The primers used were as listed in Table 1 and the amplifications were carried out under the following conditions; denaturation at 94°C for 5 seconds, annealing at 59°C for 20 seconds (group C and quadruplex) and 57°C for 20 seconds (group E), amplification at 72°C for 1 minute, and a final extension at 72°C for 5 minutes (CFX96, Bio Rad) (Table 1).

### Detection of virulence genes

The detection of the virulence genes was assessed by a list of primers adopted from previously published sequences. The oligonucleotide primers used to amplify these genes are listed in Table 2. Amplification reactions of bacterial DNA were performed in a total volume of 25μl reaction mixture containing 5μl of PCR master mix (PCR Bio), 0.5μl of each forward primer and reverse primer, 2μl of DNA template and distilled water. PCR amplifications were carried out using CFX96 Bio Rad thermocycler and the cyclic conditions were as follows: 5 minutes initial denaturation at 95°C, followed by 30 cycles of 1 minute at 94°C, 1 minute of annealing as per Table 2, 30 seconds at 72°C; with final extension step at 72°C for 5 minutes. The PCR products were analysed by electrophoresis on 1.5% agarose gel, stained with SYBR safe (Invitrogen). The gels were visualized using UV trans-illumination imaging system.

**Table 2:** List of Primers used in virulence genotyping.

Virulence genes		Primer sequence	Amplicon size (bp)	Reference
**Adhesion**				
P fimbriae	*papC*	F:GACGGCTGTACTGCAGGGTGTGGC	328	[27]
		R:ATATCCTTTCTGCAGGGATGCAATA		
Type 1 fimbriae	*fimH*	F:TGCAGAACGGATAAGCCGTGG	508	[37]
		R:GCAGTCACCTGCCCTCCGGTA		
S fimbriae	*sfa*	F:CTCCGGAGAACTGGGTGCATCTTAC	410	[38]
		R:CGGAGGAGTAATTACAAACCTGGCA		
**Iron Acquisition**				
Salmochelin receptor	*iroN*	F:AAGTCAAAGCAGGGGTTGCCCG	665	[27]
		R:GACGCCGACATTAAGACGCAG		
Yersiniabactin receptor	*fyuA*	F:TGAGTGGGAAATACACCACC	715	[39]
		R:TTACCCGCATTGCTTAATGTC		
**Toxin**				
Alpha hemolysin	*hlyA*	F:AACAAGGATAAGCACTGTTCTGGCT	1177	[37]
		R:ACCATATAAGCGGTCATTCCCGTCA		
Cytotoxic necrotising factor	*cnf*	F:AAGATGGAGTTTCCTATGCAGGAG	498	[40]
		R:TGGAGTTTCCTATGCAGGAG		
Secreted autotransporter toxin	*sat*	F:TATCACGCAATGCCAATGTT	393	[41]
		R:GACCCGGCGTTACAGTTTTA		
Uropathogen specific protein	*usp*	F:ACATTCACGGCAAGCCTCAG	448	[42]
		R:GCGAGTTCCTGGTGAAAGC		
**Capsule synthesis**				
kpsMT group II capsule	*kpsMTII*	F:GCGCATTTGCTGATACTGTTG	272	[37]
		R:CATCCAGACGATAAGCATGAGCA		
Outer membrane protease T	*ompT*	F: ATCTAGCCGAAGAAGGAGGC	559	[42]
		R:CCCGGGTCATAGTGTTCATC		

### Gene expression analysis

Extraction and purification of total RNA from two selected ASB isolates, EC095 (“high pathogenic potential strain” harbouring 10 of the 12 UPEC associated virulence genes screened in this study) and EC114 (“low pathogenic potential strain” that does not harbour any of the 12 virulence genes studied), grown to mid-logarithmic phase culture in Luria-Bertani (LB) broth were performed using RNA extraction kit (Favorgen, Biotech Corp, Taiwan) according to the manufacturer guideline. Contaminating genomic DNA was eliminated by DNase digestion kit (Favorgen, Biotech Corp, Taiwan) according to manufacturer instructions. The RNA concentration, purity, and RNA integrity were determined using a nanospectrophotometer (Nano-Drop Technology. Inc., Wilmington, DE, USA) and Agilent 2100 Bioanalyzer (Agilent Technologies, USA) respectively according to the manufacturer instruction.

#### Microarray analysis

A comparative analysis was performed between strains EC095 and EC114 to identify differentially expressed virulence genes. The expression profile of these isolates was compared using the microarray method. The samples with high RNA integrity numbers (RIN) above nine representing the biological replicates were used to prepare labelled cRNA using Low Input Quick Amp Labelling kit (Agilent Technologies, USA) following manufacturer instructions. The cRNA samples were hybridized in the Agilent Sureprint *E*. *coli* 8 X 60K microarray according to manufacturer’s protocols and subsequently scanned with Agilent microarray G2565 array laser scanner (Agilent technology). The signal intensity data were extracted using Feature Extraction Software (Version 10.7.3.1 Agilent Technologies) and the expression data were then normalised using Agilent GeneSpring Analysis Software version 14.9.1. Genes with log_2_ fold change of greater than +2 or less than -2 and adjusted p-value (Benjamini-Hochberg adjustment) of less than 0.05 were considered to be with a significant differential expression.

#### Quantitative real-time PCR

Gene expression data for *sfa*, *fyuA*, *cnf*, *hlyA*, *sat*, *kpsMT*, and *usp* genes were determined using RT-PCR, as the probes for these genes were not available in the microarray. The microarray data were also validated using the quantitative RT-PCR for the virulence genes included in this study.

### Antibiotic susceptibility testing

The recovered *E*. *coli* isolates were assessed for their antimicrobial resistance using Kirby–Bauer disc diffusion method on Mueller–Hinton agar (Hi-Media, India) following Clinical and Laboratory Standards Institute guidelines (CLSI), 2017 [43]. The following antimicrobial agents were used: ampicillin (AMP) (10μg), amoxicillin/clavulanate (AMC) (20/10μg), ampicillin/sulbactam (10/10 μg) (SAM), piperacillin/tazobactam (TP) (100/10μg), trimethoprim-sulfamethoxazole (SXT) (1.25/23.75 μg), cefuroxime (CXM) (30μg), cefoxitin (FOX) (30μg), cefotaxime (CTX) (30μg), cefepime (FEP) (30μg), ceftriaxone (CRO) (30μg), aztreonam (ATM) (30μg), imipenem (IPM) (10μg), amikacin (AK) (30μg), gentamicin (CN) (30μg) and norfloxacin (NOR) (30μg). All the antibiotic discs were procured from Oxoid, UK. The susceptibility of the *E*. *coli* isolates to each antimicrobial agent was measured and the results were interpreted based on the CLSI guidelines. *E*. *coli* ATCC 25922 was used as a quality control organism [44, 45]. A strain was defined as MDR when it exhibited resistance to a minimum of three of the antibiotic classes tested [44–46].

### Statistical analysis

The IBM Statistical Package for Social Science (SPSS) software version (26.0) was used for statistical analysis. The Pearson Chi-square and Kruskal-Wallis tests were used to find the association of phylogroups with the occurrence of virulence genes and antibiotic resistance. A p-value less than 0.05 was considered to be statistically significant.

#### Hierarchical clustering

Hierarchical clustering was performed using (SPSS Version 26.0) which results in grouping based on the presence or absence of the selected genes in the gene cluster, effectively creating a typing concurrent to the phylogenetic typing and functioning as a validity check. The four UPEC virulence functional groups of adhesion (*fimH*, *papC*, and *sfa*), iron uptake (*iroN* and *fyuA*), toxin (*hlyA*, *cnf*, *sat* and *usp*), and capsule synthesis (*ompT* and *kpsMTII*) were regarded as variables and plotted based on the presence and absence of selected virulence genes. The isolates were clustered according to the Agglomeration Schedule to plot a Dendrogram. The cluster method used in this study was ‘between-group linkage’, allowing the samples to cluster on an average distance of similarity between all pairs of two cluster members. The hierarchical clusters were grouped by measuring the Squared Euclidean Distance, thereby being able to measure the regular distance between two points in a cluster [47].

## Results

Among the 1315 urine samples screened from asymptomatic bacteriuria cases from pregnant women, 212 (16.1%) urine samples were positive for significant bacteriuria (≥ 10^5^ CFU/mL), 946 (71.9%) urine samples yielded no growth, 101 (7.6%) urine samples yielded non-significant growth and 56 (4.3%) of the urine samples were contaminated. Among the 212 samples showing significant bacteriuria, 160 (75.47% of total significant bacteriuria samples) urine culture isolates were identified as *E*. *coli*.

### Phenotypic characteristics of recovered *E*. *coli* isolates

*E*. *coli* demonstrated lactose fermenting pink colonies on MacConkey’s agar and appeared as colonies with metallic green sheen on EMB agar. Biochemically, all the isolates were positive for catalase, indole, and Methyl red, while negative for oxidase, citrate and Voges-Proskauer.

All the 160 isolates included in this study were further confirmed as *E*. *coli* based on the amplification of 16s rRNA gene for *E*. *coli*.

### Distribution of phylogroups

Overall, 55 strains belonged to phylogroups B2 (34.4%), 35 to phylogroup A (21.9%), 27 to phylogroup B1 (16.9%), 13 each to phylogroups D and E (8.1%), five strains each to phylogroups C and F (3.1%) and three strains to clade I (1.9%). Only four *E*. *coli* strains in this study were identified as non-typeable (NT) to any phylogenetic group using the extended classification. In total, 86 (53.72%) of the isolates were from the ExPEC phylogroups (B2, D, E, and F) and 74 (46.28%) from the commensals phylogroups (A, B1, C, and clade 1).

### Distributions of virulence genes

Among the 12 UPEC associated virulence genes; 11 virulence genes as depicted in Table 2 and *chuA* gene (used in phylogenetic classification from Table 1) representing four virulence functional groups were detected by amplifying the corresponding genes. Among adhesion associated virulence genes tested, occurrence of the *fimH* was the most common, followed by *papC* (20.6%) and *sfa* (16.0%) (Table 3). Regarding the iron-uptake determinants studied, the *chuA* gene was the most common (55.0%) followed by *fyuA* (31.8%) and *iroN* (24.3%). *hlyA* gene was the most common among the toxin genes, followed by *cnf* (22.5%) and *sat* (7.5%). The capsule gene *kpsMTII* showed an occurrence rate of 38.1% in the isolates studied and *ompT* in 15% of the isolates. The UPEC specific protein *usp* was found in 34.3% of the strains. All the virulence genes studied were found in different strains of phylogroup B2 and E in different permutations and combinations. Phylogroup A lacked the gene *chuA*, B1 lacked the genes *chuA* and *sat*, D lacked *sfa* and *cnf*, and phylogroup C lacked *chuA*, *iroN*, and *cnf*. Phylogroup F showed the presence of seven virulence genes except for *sfa*, *iroN*, *cnf*, and *ompT*. Clade 1 had only five virulence genes *fimH*, *iroN*, *chuA*, *fyu*, and *cnf* (Table 3). Individually, none of the 160 bacterial strains were found to harbour all 12 virulence genes included in this study.

**Table 3:** Distributions of virulence genes among ASB *E*. *coli* isolates.

Virulence gene	A	B1	B2	C	D	E	F	Clade1	NT	Total	p-value	X^2^ value
	n = 35	n = 27	n = 55	n = 5	n = 13	n = 13	n = 5	n = 3	n = 4	n = 160		
**Adhesion**												
*fimH*	27(77.1)	24(88.9)	38(69.1)	4(80.0)	9(69.2)	9(69.2)	5(100.0)	2(66.7)	3(75.0)	121 (75.6)	0.634	6.266
*sfa*	3(8.6)^b^	2(7.4)^b^	19 (34.5)^a^	2(40.0)^a^	0^b^	1(7.7)^b^	0^b^	0^b^	0^b^	27(16.9)	0.005	23.451
*papC*	5(14.3)	3(11.1)	16(29.1)	3(60.0)	3(23.1)	1(7.7)	1(20.0)	0	1(25.0)	33(20.6)	0.156	11.698
**Siderophores**												
*iroN*	5(14.3)^a^^b^	5(18.5)^a^^b^	23 (41.8)^a^	0^b^	3 (23.1)	1(7.7)^b^	0^b^	1(33.3)^a^^b^	1(25.0)^a^^b^	39(24.4)	0.030	16.843
*fyuA*	8 (22.9)	4 (14.8)	25 (45.5)	2 (40.0)	3 (23.1)	3 (23.1)	3 (60.0)	1 (33.3)	2 (50.0)	51 (31.9)	0.100	13.109
*chuA*	0^c^	0^c^	55(100)^a^^b^	0^c^	13(100)^a^^b^	13(100)^a^	5(100)^a^	0(0)^c^	2(50)^b^	88 (55.0)	0.001	110.922
**Toxin**												
*cnf*	4 (11.1)^b^	4(14.8)^b^	24 (43.6)^a^	0^b^	0^b^	2 (15.4)^a^^b^	0^b^	1 (33.3)^a^^b^	1 (25.0)^a^^b^	36 (22.5)	0.002	24.737
*hlyA*	6 (17.1)^a^^b^	8 (29.6)^a^^b^	25 (45.5)^a^	1 (20.0)^a^^b^	2 (15.4)^a^^b^	1 (7.7)^b^	1 (20.0)^a^^b^	0^b^	0^b^	44 (27.5)	0.024	17.290
*sat*	4 (11.4)^a^^b^^c^	0^c^	1 (1.8)^b^^c^	2 (40.0)^a^^b^	1 (7.7)^a^^b^^c^	2 (15.4)^a^^b^^c^	2 (40.0)^a^	0^b^^c^	0^b^^c^	12 (7.5)	0.015	22.486
*usp*	3 (8.6)^b^	2 (7.4)^b^	38 (69.1)^a^	1 (20.0)^b^	4 (30.8)^b^	3 (23.1)^b^	1 (20.0)^b^	0^b^	3 (75.0)^a^	55 (34.4)	<0.001	54.643
**Capsule synthesis**												
*kpsMTII*	5 (14.3)^b^	2 (7.4)b	36 (65.5)^a^	2 (40.0)^a^^b^	5 (38.5)^a^^b^	4 (30.8)^a^^b^	4 (80.0)^a^	0^b^	3 (4.9)^a^	61 (38.1)	<0.001	44.823
*ompT*	3 (8.6)	3 (11.1)	7 (12.7)	1 (20.0)	3 (23.1)	5 (38.5)	0	0	2 (50.0)	24 (15.0)	0.101	13.308

^a,b,c^ Group with different alphabets are statistically different at 0.05 level based on pairwise comparison.

Overall, the occurrence of 12 virulence genes in this study was higher among isolates from phylogroup B2. The statistical analysis using the Chi-square test demonstrated that there is a significant difference (p<0.05) in the occurrence of eight virulence genes (*sfa*, *chuA*, *iroN*, *cnf*, *hlyA*, *usp*, *sat*, and *kpsMTII*) (Table 3) among phylogroups. Analysis of the association in the presence of virulence genes with phylogroups using Kruskal-Wallis tests demonstrated a statistically significant higher presence of *sfa*, *iroN*, *cnf*, *hlyA*, and *usp* virulence genes with phylogroup B2 than other phylogroups. The presence of the *chuA* virulence gene was only associated with ExPEC associated phylogroups.

### Gene expression analysis

The gene expression data from microarray and RT-PCR analysis revealed that strain EC095 (termed as “high pathogenic potential strain” as it harbours 10 UPEC associated virulent genes screened in this study) expressed 9/12 virulence genes (*fimH*, *papC*, *iroN*, *fyuA*, *chuA*, *cnf*, *hlyA*, *ompT*, *and kpsMTII*) (Table 4). Although *usp* was detected to be present in the genome of EC095, it did not show expression. As expected, none of the 12 genes studied were expressed in strain EC114 (termed as “low pathogenic potential strain” as it does not harbour any of the virulence genes studied).

**Table 4:** Summary of the gene expression profile of two selected ASB isolates.

		*Adhesion*			*Iron uptake*			*Toxin*			*Capsule synthesis*		
		*fimH* *	*Sfa* **	*papC* *	*iroN* *	*fyuA* **	*chuA* *	*cnf* **	*hlyA* **	*sat* **	*ompT* *	*kpsMTII* **	*usp* **
EC114 (low pathogenic potential)	Conventional PCR (DNA)	-	-	-	-	-	-	-	-	-	-	-	-
	Gene expression (RNA)	-	-	-	-	-	-	-	-	-	-	-	-
EC095 (high pathogenic potential)	Conventional PCR (DNA)	+	-	+	+	+	+	+	+	-	+	+	+
	Gene expression (RNA)	+	-	+	+	+	+	+	+	-	+	+	-

*Gene expression profiles obtained from microarray analysis.

**Gene expression profiles obtained from RT-PCR.

+ indicates the presence of expression.

- indicates the absence of expression.

Interestingly, the result for differentially expressed genes (DEG) obtained from microarray analysis revealed that the expression of *fimH*, *papC*, *iroN*, *chuA*, *hlyA*, and *kpsMT* gene were not only upregulated for the particular genes included in this study, but the entire gene clusters of the respective genes were also significantly upregulated (Table 5). For the *usp* and *omp* gene clusters, the expression of two regulator genes (*ompR* and *uspC*) were identified as down-regulated in EC095 strain (Table 5).

**Table 5:** Significant differentially expressed virulence genes among ASB EC095 (high pathogenic potential) strain compared to EC114 (low pathogenic potential) strain using microarray analysis.

Gene	Regulation	Fold change	Description
*fimA*	Up	4.0393	major type 1 subunit fimbrin
*fimB*	Up	3.3423	recombinase involved in phase variation; regulator for fimA
*fimC*	Up	5.3053	periplasmic chaperone, required for type 1 fimbriae
*fimD*	Up	3.1863	export and assembly of type 1 fimbriae
*fimE*	Up	4.7253	recombinase involved in phase variation; regulator for fimA
*fimF*	Up	2.7634	fimbrial morphology
*fimG*	Up	2.9985	fimbrial morphology
*fimH* *	Up	5.4889	minor fimbrial subunit, D-mannose specific adhesin
*fimI*	Up	7.2923	fimbrial protein
*papC* *	Up	3.4937	PapC protein
*papF*	Up	2.8650	PapF protein
*papG*	Up	4.2843	PapG protein
*papJ*	Up	2.9173	PapJ protein
*papK*	Up	4.7184	PapK protein
*papX*	Up	6.3801	PapX protein
*sfaB*	Up	5.1316	Putative F1C and S fimbrial switch Regulatory
*iroB*	Up	4.6443	Putative glucosyltransferase
*iroD*	Up	4.1750	Ferric enterochelin esterase
*iroE*	Up	5.0085	IroE protein
*iroN* *	Up	5.2658	Siderophore receptor IroN
*chuA* *	Up	3.2172	Outer membrane heme/hemoglobin receptor
*chuS*	Up	2.1689	putative heme/hemoglobin transport protein
*chuT*	Up	3.7738	Putative Periplasmic binding protein
*chuU*	Up	3.6617	Putative permease of iron compound ABC transport
*chuW*	Up	2.2960	Putative oxygen-independent coproporphyrinogen III oxidase
*chuX*	Up	2.1485	Hypothetical protein
*chuY*	Up	3.2964	Hypothetical protein
*hlyB*	Up	5.0457	Hemolysin B
*hlyC*	Up	4.8671	Hemolysin C
*hlyD*	Up	5.3834	Hemolysin D
*ompF*	Up	2.0408	outer membrane protein
*ompG*	Up	2.2054	outer membrane protein
*ompR*	Down	-2.7222	response regulator
*ompT* *	Up	6.2844	Protease VII precursor
*ompW*	Up	1.7231	Outer membrane protein W precursor
*kpsC*	Up	2.0198	KpsC protein
*kpsD*	Up	3.6400	KpsD protein
*kpsE*	Up	2.0873	KpsE protein
*uspB*	Down	-1.2465	orf, hypothetical protein
*uspC*	Down	-1.7622	putative regulator
*usG*	Up	11.6176	orf, hypothetical protein

All the genes included in this table were with log2 fold change of greater than two or less than −2 and with adjusted *p*-value (Benjamini-Hochberg adjustment) of less than 0.05 which showed significant differential expression.

* Virulence genes of interest in this study.

### Resistance to antibiotics

*E*. *coli* isolated in this study were tested against 15 antibiotics from different antimicrobial classes such as penicillin, penicillin with beta-lactamase inhibitors, antipseudomonal with beta lactamase inhibitors, non-extended spectrum cephalosporin, extended spectrum cephalosporins, cephamycin, folate pathway inhibitors, monobactam, carbapenem, aminoglycoside, and fluoroquinolones (Table 6). The examined strains revealed a higher resistance level to AMP (74.3%) followed by AMC (54.4%) among the antibiotics tested. The resistance to other antimicrobial agents observed were SXT (46.9%), CXM (33.8%), SAM (32.5%), TP (28.7%), CTX (26.3%), IPM (24.4%), CRO (21.9%), FEP (19.4%), FOX (15.6%), ATM (15.0%), CN (11.3%) and NOR (5.6%) in the descending order.

**Table 6:** Antibiotic resistance profile of *E*. *coli* among different phylogroups.

	Group A	Group B1	Group B2	Group C	Group D	Group E	Group F	Clade 1	Unknown	Total	p-value *
	n = 35	n = 27	n = 55	n = 5	n = 13	n = 13	n = 5	n = 3	n = 4	n = 160	
**Penicillin**											
Ampicillin	26 (74.3)	23 (85.2)	43 (78.2)	3 (60.0)	11 (84.6)	10 (76.9)	2 (40.0)	2 (66.7)	4 (100.0)	124 (77.5)	0.454
**Penicillin with β-lactamase inhibitors**											
Amoxicillin-clavulanate	16 (45.7)	20 (74.1)	24 (43.6)	2 (40.0)	8 (61.5)	11 (84.6)	2 (40.0)	2 (66.7)	2 (50.0)	87 (54.4)	0.083
Ampicillin-Sulbactam	12 (34.3)	12 (44.4)	14 (25.5)	2 (40.0)	5 (38.5)	6 (46.2)	0	1 (33.3)	0	52 (32.5)	0.357
**Antipdeudomonal with β-lactamase inhibitors**											
Piperacillin- Tazobactam	11 (31.4)	8 (29.6)	12 (21.8)	0	4 (30.8)	7 (53.8)	1 (20.0)	1 (33.3)	2 (50.0)	46 (28.7)	0.381
**Non-extended spectrum cephalosporins (1**^**st**^ **and 2**^**nd**^ **generation)**											
Ceforoxime	13 (37.1)	11 (40.7)	15 (27.3)	0	7 (53.8)	5 (38.5)	1 (20.0)	1 (33.3)	1 (25.0)	54 (33.8)	0.496
**Extended spectrum cephalosporins (3**^**rd**^ **and 4**^**th**^ **generation)**											
Cefotaxime	10 (28.6)	8 (29.6)	14 (25.5)	1 (20.0)	2 (15.4)	3 (23.1)	1 (20.0)	1 (33.3)	2 (50.0)	42 (26.3)	0.958
Cefepime	9 (25.7)	5 (18.5)	8 (14.5)	0	3 (23.1)	5 (38.5)	1 (20.0)	0	0	31 (19.4)	0.457
Ceftriaxone	10 (28.6)	8 (29.6)	9 (16.4)	0	3 (23.1)	3 (23.1)	1 (20.0)	1 (33.3)	0	35 (21.9)	0.689
**Cephamycin**											
Cefoxitin	8 (22.9)	5 (18.5)	6 (10.9)	0	2 (15.4)	3 (23.1)	1 (20.0)	0	0	25 (15.6)	0.722
**Folate pathway inhibitor**											
Trimethprim -sulfamethoxazole	20 (57.1)	14 (51.9)	19 (34.5)	3 (60.0)	7 (53.8)	7 (53.8)	3 (60.0)	0	2 (50.0)	75 (46.9)	0.356
**Monobactam**											
Aztreonam	6 (17.1)	5 (18.5)	7 (12.7)	0	1 (7.7)	2 (15.4)	1 (20.0)	1 (33.3)	1 (25.0)	24 (15.0)	0.918
**Carbapenem**											
Imipenem	11 (31.4)	4 (14.8)	14 (25.5)	0	4 (30.8)	5 (38.5)	0	1 (33.6)	0	39 (24.4)	0.373
**Aminoglycoside**											
Amikacin	15 (42.9)	8 (29.6)	23 (41.8)	3 (60.0)	7 (53.8)	8 (61.5)	2 (40.0)	2 (66.7)	2 (50.0)	70 (43.8)	0.674
Gentamicin	5 (14.3)	4 (14.8)	5 (9.1)	0	0	4 (30.8)	0	0	0	18 (11.3)	0.288
**Fluoroquinolones**											
Norfloxacin	5 (14.3)	3 (11.1)	1 (1.8)	0	0	0	0	0	0	9 (5.6)	0.229
MDR	18 (51.4)	15 (55.6)	25 (45.5)	2 (40.0)	8 (61.5)	10 (76.9)	1 (20.0)	2 (66.7)	2 (50.0)	83 (51.9)	
Non MDR	16 (45.7)	12 (44.4)	30 (54.5)	3 (60.0)	5 (58.5)	3(23.1)	4 (80.00)	1 (33.3)	2 (50.0)	77 (38.1)	

* There is no significant difference identified between antibiotic tested and phylogroups.

The distribution of resistant strains among phylogroups revealed that *E*. *coli* from phylogroups A, B1, and B2 were resistant to all antibiotic classes tested in this study. It was also observed that *E*. *coli* from phylogroups E showed resistance to 14 antibiotics except NOR and isolates from phylogroup D showed resistance to 13 antibiotics except CN and NOR. The other phylogroups (C, F and clade 1) showed resistance to the lesser extent of tested antibiotics. Overall, there was no statistical difference observed in the distribution of resistance among *E*. *coli* from different phylogroups. Analysis of the occurrence of MDR in this study revealed that 83 (51.9%) *E*. *coli* isolates were MDR. Among the MDR isolates, 25 were from phylogroups B2, 18 from group A, 15 from group B1, 10 from group E, eight from group D, two strains from group C and clade 1, and only one strain from group F.

Correlation analysis among the tested antimicrobial agents did not reveal any strong positive correlation (r = 0.90–1.00) between the tested antibiotics (Table 7). However, there was a low to moderate correlation observed between AMP, AMC, SAM, TP, CXM, FOX, CTX, FEP, CRO, ATM, IPM and NOR (with a correlation significant at 0.01 level). Moreover, there is no correlation observed between AK and AMP (r = 0.113); AK and AMC (r = 0.1); AK and SAM (r = 0.141), AK and CXM (r = 0.143); AK and FOX (r = 0.037), and AK and CTX (r = 0.132). Besides, NOR also showed no correlation between AMP (r = 0.067), SAM (r = 0.062), FEP (r = 0.018) and CN (r = 0.085). Overall, there were only two negative correlations identified, between NOR and TP (r = 0.035) and NOR and IPM (r = 0.102).

**Table 7:** The correlation between the tested antimicrobial agents.

	AMP	AMC	SAM	TP	CXM	FOX	CTX	FEP	CRO	SXT	ATM	IPM	AL	CN	NOR
AMP	1														
AMC	.558**	1													
SAM	.374**	.475**	1												
TP	.309**	.388**	.444**	1											
CXM	.226**	.309**	.323**	.335**	1										
FOX	.232**	.291**	.473**	.335**	.312**	1									
CTX	.287**	.318**	.405**	.374**	.415**	.408**	1								
FEP	.188*	.259**	.234**	.387**	.386**	.225**	.319**	1							
CRO	.285**	.394**	.343**	.566**	.582**	.314**	.509**	.582**	1						
SXT	.332**	.378**	.401**	.356**	.226**	.338**	.222*	.326**	.290**	1					
ATM	.226**	.279**	.344**	.507**	.478**	.205**	.386**	.370**	.582**	.239**	1				
IPM	.236**	.315**	.414**	.476**	.334**	.397**	.224**	.348**	.369**	.465**	.332**	1			
AK	0.113	0.1	0.141	.303**	0.143	0.037	0.132	.205**	.295**	.534**	.194*	.233**	1		
CN	.192*	.167*	.344**	.386**	.206**	.337**	.192*	.326**	.290**	.743**	.404**	.443**	.164*	1	
NOR	.067	.169*	.062	-0.035	.342**	.194*	.224**	0.018	.265**	.364**	.201*	-0.012	.161*	0.085	1

** Correlation is significant at the 0.01 level (2-tailed),

* Correlation is significant at the 0.05 level (2-tailed).

### Hierarchical clustering

**Fig 1:**
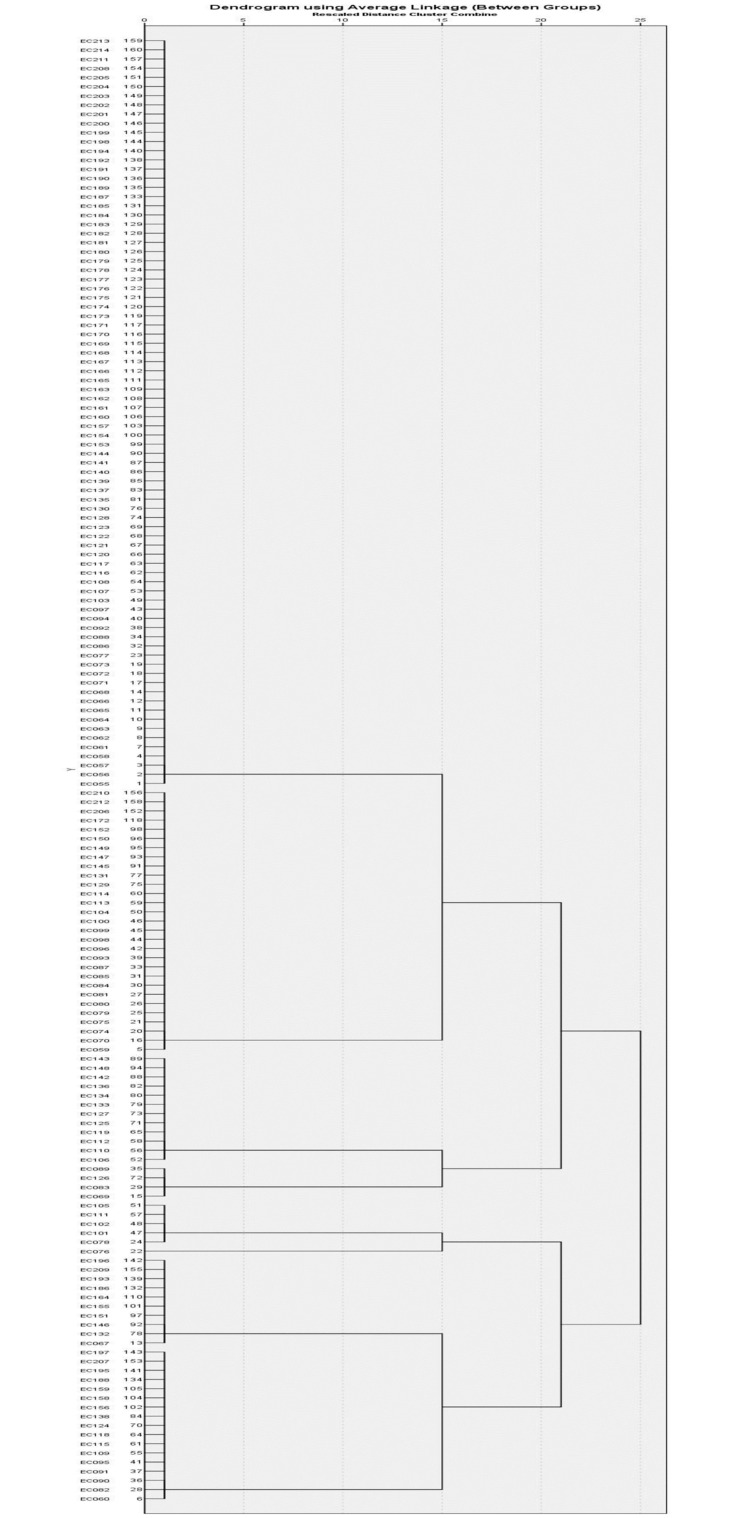
Hierarchical clustering of *E*. *coli* isolates according to the presence of adhesion functional virulence genes (*fimH*, *papC* and *sfa*).

Hierarchical clustering analysis was performed separately for each of the four virulence functional groups to deduce the distribution of virulence genes among the phylogroups. Cluster analysis for the adhesion virulence functional group (*fimH*, *papC*, and *sfa*) revealed that *E*. *coli* isolated from ASB were clustered into five groups based on the presence or absence of virulence genes responsible for adhesion (Table 8, Fig 1). The biggest cluster identified was cluster I, whereby 81 (50.6%) of the ASB isolates harboured only *fimH* gene in its genome. Isolates from cluster II had two adhesion genes (*fimH* and *sfa*) and isolates from cluster V had two genes (*papC* and *fimH*). The study identified only 10 (6.3%) strains that had all three adhesion genes (*fimH*, *papC*, and *sfa*) tested in this study (Cluster IV). Among these isolates, eight strains were from phylogroup B2 and one strain from phylogroup A and B1, respectively. Twenty-nine (18.1%) of the ASB isolates did not harbour any of the adhesion genes tested in this study (Cluster III) and the majority of these isolates were from the ExPEC phylogroup B2.

**Table 8:** Hierarchical clustering for distribution of adhesion virulence genes among *E*. *coli* isolated from ASB cases, total (%).

	Cluster I	Cluster II	Cluster III	Cluster IV	Cluster V	Non clusterable
	81 (50.6)	11 (6.9)	29 (18.1)	10 (6.3)	19 (11.9)	10 (6.3)
**Virulence gene**						
*fimH*	81 (66.9)	11 (9.1)	0	10 (8.3)	19 (15.7)	0
*papC*	0	0	0	10 (30.3)	19 (51.5)	6 (18.2)
*sfa*	0	11 (40.7)	0	10 (37.0)	0	6 (18.5)
**Phylogroups**						
Group A	21 (25.9)	1 (9.10)	6 (20.7)	1 (10.0)	4 (21.1)	2 (20.0)
Group B1	20 (24.7)	1 (9.1)	3 (10.3)	1 (10.0)	2 (10.5)	0
Group B2	18 (22.2)	8 (72.7)	11 (37.9)	8 (80.0)	4 (21.4)	6 (60.0)
Group C	0	0	0	0	4 (21.1)	1 (10.0)
Group D	7 (8.6)	0	3 (10.3)	0	2 (10.5)	1 (10.0)
Group E	7 (8.6)	1 (9.1)	4 (13.8)	0	1 (5.3)	0
Group F	4 (4.9)	0	0	0	1 (5.3)	0
Clade 1	2 (2.5)	0	1 (3.4)	0	0	0
NT	2 (2.5)	0	1 (3.4)	0	1 (5.30)	0

**Fig 2:**
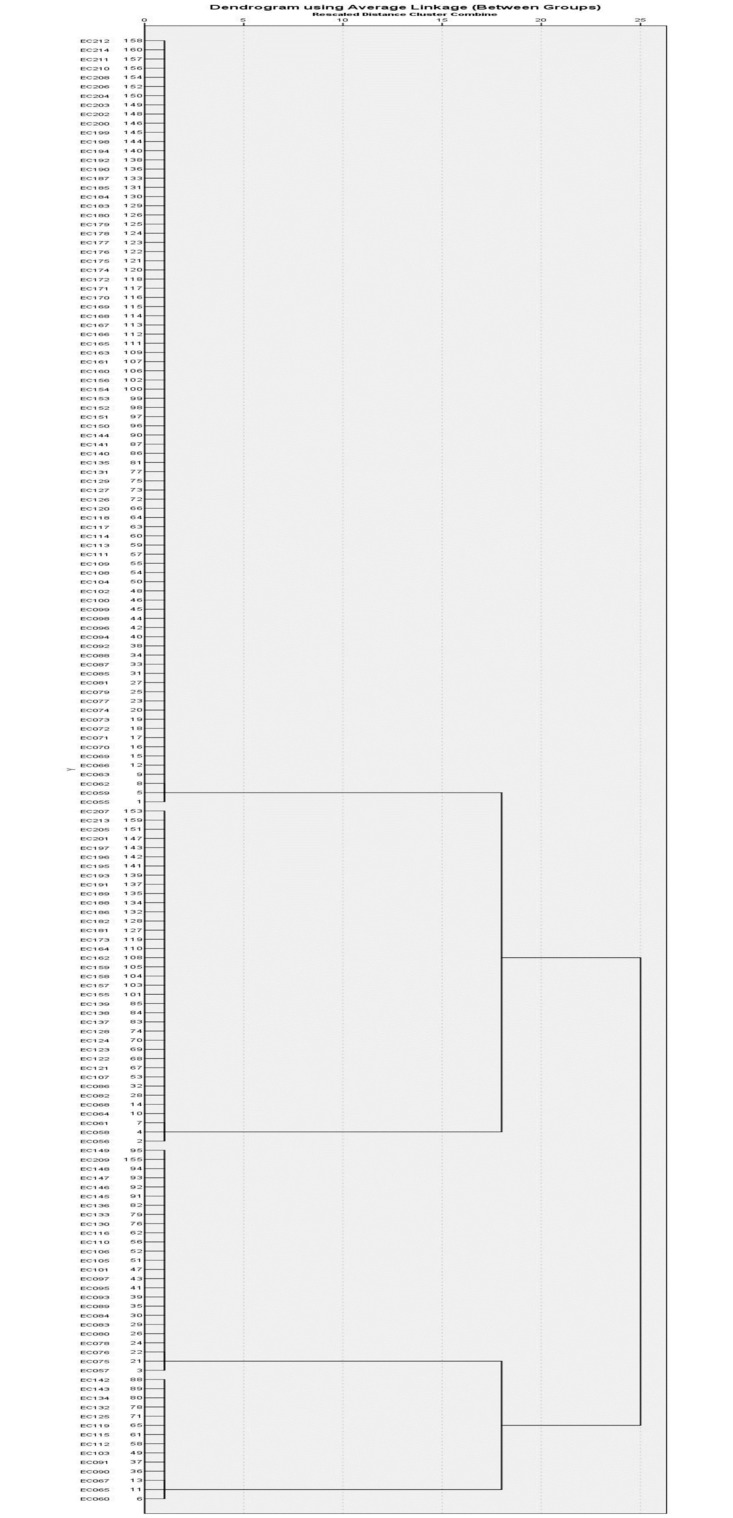
Hierarchical clustering of *E*. *coli* isolates according to the presence of iron uptake functional virulence genes (*iroN* and *fyuA*).

Hierarchical clustering according to the presence and absence of iron uptake related virulence genes (*fyuA* and *iroN*), grouped the isolates into four clusters (Table 9, Fig 2). The majority (52.5%) of the isolates were grouped into Cluster I and did not have both the iron uptake associated virulence genes tested in this study. Isolates from this null cluster were distributed among all phylogroups. Fourteen (8.8%) isolates were in Cluster III containing both the *fyuA* and *iroN* genes, among which ten (71.4%) belonged to phylogroup B2. There were 25 (15.6%) isolates in Cluster II and 37 (23.1%) in Cluster IV containing only *iroN* and *fyuA* genes, respectively. It is to be noted that the *chuA* gene, with the highest distribution (58.7%) among the iron uptake related virulence genes (Table 3) was not used in this clustering as this gene is also one of the genetic markers used in phylogenetic classification. The use of this gene as a variable in cluster analysis will also lead to clustering similar to the phylogroups.

**Table 9:** Hierarchical clustering for distribution of iron acquisition virulence genes among *E*. *coli* isolated from ASB cases, total (%).

	Cluster I	Cluster II	Cluster III	Cluster IV
	84 (52.5)	25 (15.6)	14 (8.8)	37 (23.1)
**Virulence gene**				
*iroN*	0	25 (64.1)	14 (35.9)	0
*fyuA*	0	0	14 (27.5)	37 (72.5)
**Phylogroups**				
Group A	23 (27.4)	4 (16.0)	1 (7.1)	7 (18.9)
Group B1	21 (25.0)	2 (8.0)	3 (21.4)	1 (2.7)
Group B2	17 (20.2)	13 (52.0)	10 (71.4)	15 (40.5)
Group C	3 (3.6)	0	0	2 (5.4)
Group D	7 (8.3)	3 (12.0)	0	3 (8.1)
Group E	9 (10.7)	1 (4.0)	0	3 (8.1)
Group F	2 (2.4)	0	0	3 (8.1)
Clade 1	1 (1.2)	1 (4.0)	0	1 (2.7)
NT	1 (1.2)	1 (4.0)	0	2 (5.4)

**Fig 3:**
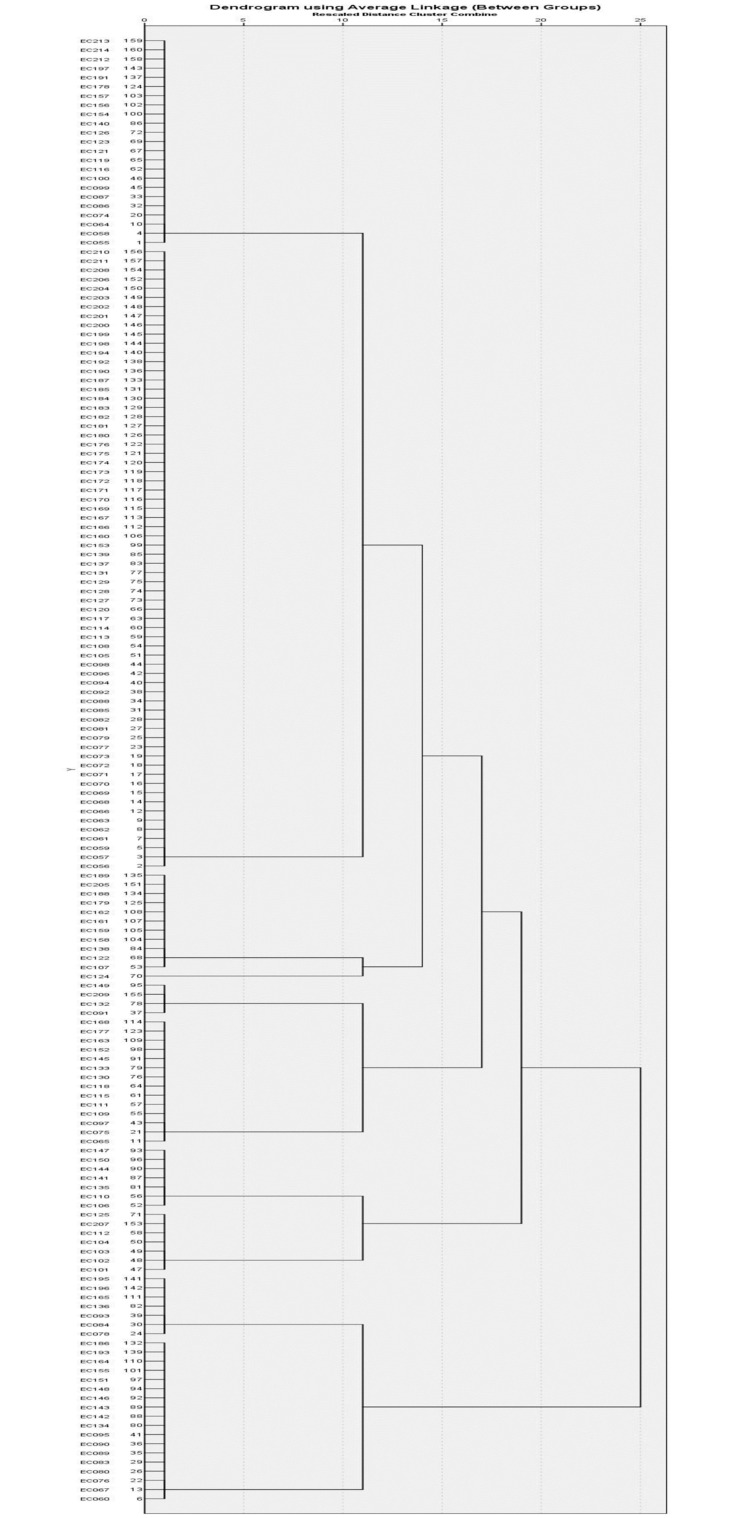
Hierarchical clustering of *E*. *coli* isolates according to the presence of toxin functional virulence genes (*cnf*, *sat*, *hlyA* and *usp*).

Overall, five clusters were identified based on the presence and absence of the toxin associated virulence genes *cnf*, *hlyA*, *sat* and *usp* (Table 10, Fig 3). Cluster II, III, and IV contained isolates with only one of the four toxin-related genes tested. Cluster V consists of 18 (11.3%) isolates that harboured three of the toxin genes (*cnf*, *hlyA*, and *usp)* in their genome. Among these isolates, 15 were from phylogroup B2 and only one strain each were from phylogroups A, B1, and E, respectively. Interestingly, none of the clusters had all four of the virulence genes tested. A total of 68 (42.5%) strains grouped in Cluster I (null cluster) did not harbour any toxin genes tested in this study. The strains from this Cluster I demonstrated a higher number of strains from phylogroups A and B1 compared to strains from other phylogroups.

**Table 10:** Hierarchical clustering for distribution of toxin virulence genes among *E*. *coli* isolated from ASB cases, total (%).

	Cluster I	Cluster II	Cluster III	Cluster IV	Cluster V	Nonclusterable
	68 (42.5)	14 (8.8)	11 (6.90)	23 (14.4)	18 (11.3)	26 (16.3)
**Virulence gene**						
*cnf*	0	0	0	0	18 (50.0)	18 (50.0)
*hlyA*	0	14 (31.8)	0	0	18 (40.9)	12 (27.3)
*sat*	0	0	11 (91.7)	0	0	1 (8.3)
*usp*	0	0	0	23 (41.8)	18 (32.7)	14 (25.5)
**Phylogroups**						
Group A	20 (29.4)	5 (35.7)	4 (36.4)	2 (8.7)	1 (5.6)	3 (11.5)
Group B1	17 (25.0)	5 (35.7)	0	1 (4.3)	1 (5.6)	3 (11.5)
Group B2	10 (14.7)	3 (21.4)	1 (9.1)	12 (52.5)	15 (83.3)	14 (53.8)
Group C	1 (1.5)	1 (7.1)	2 (18.2)	1 (4.3)	0	0
Group D	8 (11.8)	0	1 (9.1)	2 (8.7)	0	2 (7.7)
Group E	7 (10.3)	0	2 (18.2)	2 (8.7)	1 (5.6)	1 (3.8)
Group F	2 (2.9)	0	1 (9.1)	1 (4.3)	0	1 (3.8)
Clade 1	2 (2.9)	0	0	0	0	1 (3.8)
NT	1 (1.5)	0	0	2 (8.7)	0	1 (3.8)

**Fig 4:**
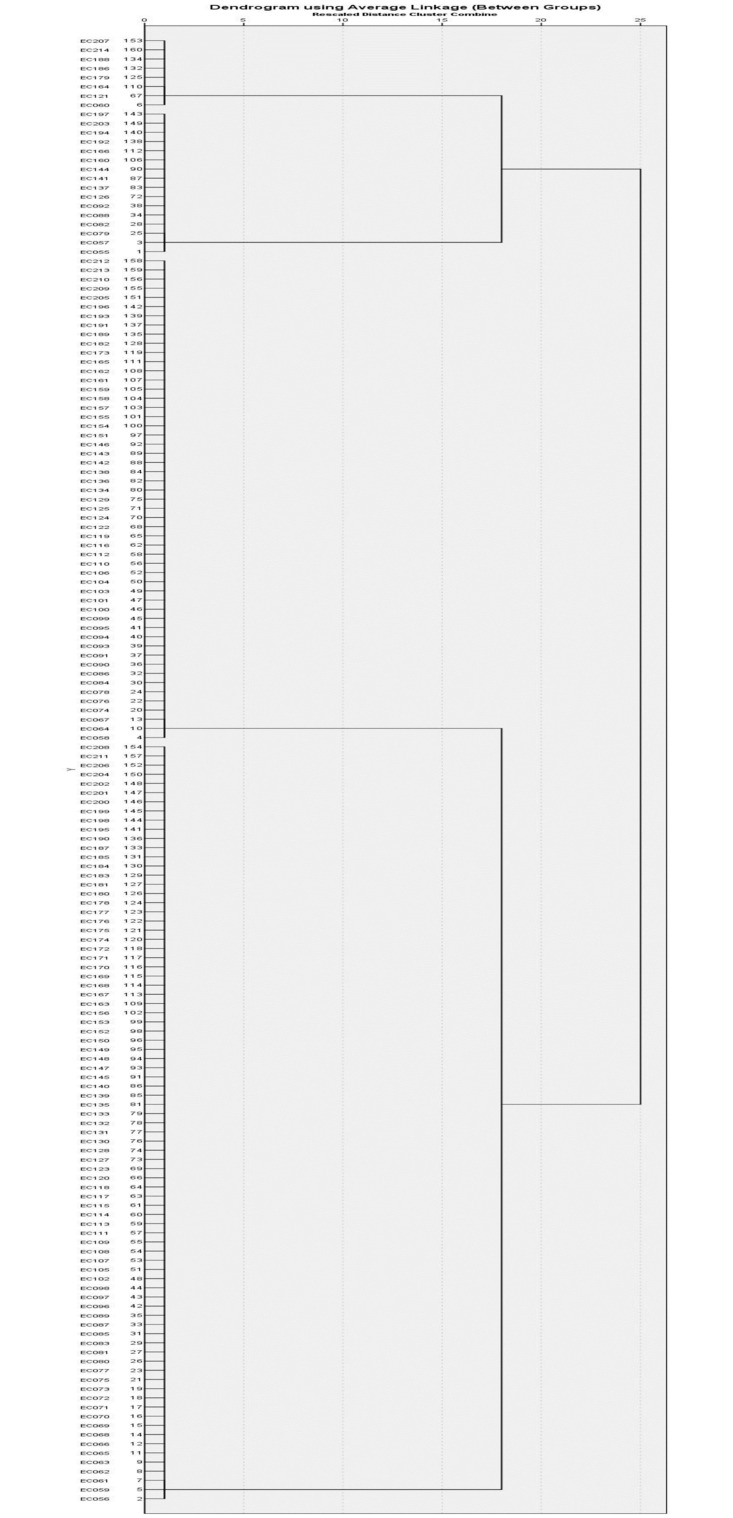
Hierarchical clustering of *E*. *coli* isolates according to the presence of capsule production functional virulence genes (*kpsMTII* and *ompT*).

Clustering of ASB isolates based on the capsule synthesis virulence functional genes (*kpsMTII* and *ompT*) identified four different clusters (Table 11, Fig 4). The biggest cluster was Cluster I, which did not have both the *kpsMTII* and *ompT* genes (null cluster). The isolates from this group were distributed higher among phylogroup A, B1 and B2 compared with other phylogroups. The second biggest cluster was Cluster IV with 52 (32.5%) strains that only acquired the *kpsMTII* virulence gene. Cluster II had 16 (10.0%) strains with the *ompT* gene. There were only 8 (5.0%) strains identified to have both capsule synthesis associated virulence genes tested in this study and five of these isolates were from phylogroup B2.

**Table 11:** Hierarchical clustering for distribution of capsule synthesis virulence genes among *E*. *coli* isolated from ASB cases, total (%).

	Cluster I	Cluster II	Cluster III	Cluster IV
	84 (52.5)	16 (10.0)	8 (5.0)	52 (32.5)
**Virulence gene**				
*kpsMT*II	0	0	8 (13.1)	52 (85.2)
*ompT*	0	16 (66.7)	8 (33.3)	0
**Phylogroups**				
Group A	27 (32.1)	3 (18.8)	0	5 (9.6)
Group B1	22 (26.2)	3 (18.8)	0	2 (3.8)
Group B2	18 (21.4)	2 (12.5)	5 (62.5)	30 (57.7)
Group C	2 (2.4)	1 (6.3)	0	2 (3.8)
Group D	5 (6.0)	3 (18.8)	0	5 (9.6)
Group E	6 (7.1)	3 (18.8)	2 (25.0)	2 (3.8)
Group F	1 (1.2)	0	0	4 (7.7)
Clade 1	3 (3.6)	0	0	0
NT	0	1 (6.3)	1 (12.5)	2 (3.8)

## Discussion

### The occurrence of ASB among pregnant women

The occurrence of ASB in this study was 16.1%, in comparison to 1.9% of significant ASB cases among pregnant women in Malaysia [33]. This could be due to the study site being a tertiary care public hospital, where most of the cases were referred, expertise was available, and also the preferred choice due to the subsidized cost. Our data revealed that the occurrence of ASB among the tested population in this study was comparatively higher than reported in Nepal (8.7%), Egypt (10.0%), Iran (11.6%), and Nigeria (14.6%) but lower than 61.5% occurrence reported in Nepal, 20–46% in Iran and 38.9% in India [4, 48–50]. These variations in the frequency of ASB cases and distribution might be attributed to the differences in the geographical area, socioeconomic status, differences in the community healthcare infrastructure, and also awareness on standards of personal hygiene practices of women who participated in the respective studies.

### Distribution of phylogroups among the isolates

Phylogenetic analysis data showed that the *E*. *coli* isolated from ASB cases in the study population were distributed into phylogroups B2, A, B1, D, E, C, F and clade I in decreasing order of frequency. The distribution of ExPEC (B2, D, E, and F) phylogroups were found to be higher than non-ExPEC (A, B1, C, and clade 1) phylogroups. This significantly higher occurrence of ExPEC among ASB indicates the possibility that these *E*. *coli* were probably attenuated strains from UPEC causing symptomatic UTI.

*E*. *coli* from phylogroup B2 and D were mostly demonstrated to be higher among UPEC isolates from symptomatic UTI cases such as cystitis, pyelonephritis, and urosepsis [38, 51–53]. The pathogenic nature of B2 and D thus has been demonstrated many times. As per Clermont ‘s extended classification [18] *E*. *coli* from phylogroup E is related to phylogroup D and phylogroup F is a subgroup of phylogroup B2. Thus, in the newer classification, *E*. *coli* from phylogroups E and F should be considered pathogenic [54–56]. Data from this study revealed that *E*. *coli* from phylogroup E and F also harboured most of the virulence genes studied, but in lower frequency. *E*. coli from the non-ExPEC phylogroups (A, B1, C and clade 1) were also found in this study. However, the virulence nature of these phylogroups can only be evaluated based on the occurrence of the virulence genes among the strains from these phylogroups.

Among the mammalian hosts, the distribution of *E*. *coli* phylogroups was shown to be different and the probable reason for this was stated to be the environmental conditions such as host habitat, climate, diet, gut morphology, use of antimicrobial agents, and their effects on commensals in fecal flora [57]. The fecal flora which later colonizes the perineal and perianal area becomes the source of bacteria infecting or colonising the urinary tract, especially in females. Phylogenetic group B2 (or the whole ExPEC), which could be more adapted for extraintestinal survival then becomes the naturally selected phylogroups to be observed in the extraintestinal sites. The question then remains would be which event occurs first, symptomatic infection or asymptomatic colonization or an outcome decided by the genetic adaptability of the colonising organisms and their interaction with the host immune system. This probably could partly be answered by understanding the distribution of virulence determinants.

### Distribution of virulence genes among the isolates

The virulence genes ensure the survival of bacteria in different challenging environments and the retention, acquisition, or loss of the gene might be because of environmental stimulus. Asymptomatic bacteriuria might indicate a commensalistic existence in usually sterile mucosal areas of the urinary tract like bladder mucosa. This most logically could progress in a long-term association without causing clinical manifestation or could result in a clinically manifesting infection. The retention or loss of virulence genes and their subsequent probable expression if retained could shift the balance of this association in either way. Although initial colonisation occurs in the majority of individuals in the community, the progression into pathogenic association would also be influenced by host susceptibility factors including comorbidities which was not the main focus of this study. The selection of 12 virulence genes in this study was made based on the rationale of higher citation and higher reported existence among UPEC generated through literature review [42, 58–60].

#### Adhesion

Among UPEC, the presence of adhesion-associated genes were reported to help bacteria to withstand the hydrodynamic force of bulk urine excretion and thus, enhance colonization in the genitourinary tract. In the present work, the frequency of the *fimH* gene (75.6%) among ASB isolates was lower compared to its frequency from previous studies on pregnancy-associated ASB isolates which ranged from 90–98% [37, 51, 61]. A similar higher distribution of *fimH* was also reported among UPEC isolated from symptomatic UTI cases [62, 63]. The acquisition of the *fimH* gene will facilitate the adherence of *E*. *coli* to a series of non-glycosylated peptide epitopes and glycoproteins in the urothelium of the urinary tract which aided its prolonged survival [64, 65].

In this study, the occurrence of the *papC* gene among the ASB isolates was found to be low (20.6%) compared to *fimH*. Our finding was also supported by a previous study which demonstrated 18.9% occurrence of *papC* in ASB isolates compared with 23.1% in cystitis and 64.3% in pyelonephritis [66]. The low prevalence of *papC* in our ASB isolates could be due to attenuation of *papC* as part of their strategy to colonize the bladder without triggering an immune response. In this study, the distribution of the *papC* gene was the highest in phylogroup B2 in comparison to other phylogroups. Considering that most UPEC causing symptomatic UTIs to belong to phylogroup B2, and evidences showing that ASB strains have evolved from UPEC by attenuating their genome, the B2 strains of this study have probably retained their *papC* gene through the process of transformation from symptomatic pathogens to asymptomatic colonizers. Similar to *papC*, the occurrence of the *sfa* gene in this study was also low. Tabasi et al. [67], also reported a similar lower distribution of this gene among ASB (12.9%).

Although the reasons for the difference in presence of this *papC* and *sfa* gene among the *E*. *coli* from ASB cases was not fully detailed in the literature, the data generally indicates a strong possibility of retention of one gene (*fimH)* and selective loss of another gene which was shown to be associated with immune response (*papC* or *sfa)*. This during the process of coexistence with the host, probably led to commensalistic existence and development of ASB in women. This data also suggests a possible virulence marker (*papC)* for identifying a potentially pathogenic UPEC among the ASB isolates which could later cause a symptomatic UTI.

#### Iron acquisition

Bacteria need access to iron to properly promote cellular processes, replicate and cause disease [56]. The urinary tract and its environment are limited in iron source, therefore *E*. *coli* has equipped themselves with several iron-acquisition genes to scavenge the iron from uroepithelial cells [68]. In addition, with limited adhesion repertoire, a significant factor that contributes to the persistence of *E*. *coli* 83972 (ASB prototype strain) in the urinary tract was found to be the enhanced siderophore production [69, 70]. The upregulation of iron uptake and transport genes in *E*. *coli* 83972, was also reported to contribute to its rapid growing ability which helps to compensate for the lack of other virulence genes associated with adhesions and toxins, thus aiding in its prolonged colonization in the urinary tract system without evoking the inflammatory response in the host [69]. Data generated in this study demonstrated that the distribution of the iron-uptake genes, *iroN* and *fyuA*, were 24.3% and 31.8%, respectively (Table 3). This study demonstrated that isolates of phylogenetic group B2 had the highest number of isolates positive for *iroN* and *fyuA*. The presence and probable utilisation of those two genes by strains belonging to phylogroup B2 would have given them an advantage over other groups in colonizing the urinary tract, and thus, increase their occurrence in the community.

Considering the UPEC strains isolated from clinical cases of UTI, aerobactin iron uptake systems were reported to be found in higher frequency among pyelonephritis (73%) and cystitis (49%) isolates of UPEC [71]. Comparative lower frequency of presence of siderophore genes, yet surviving in the urinary tract could be with the help of other iron uptake systems which was not addressed in this study. The analysis of the data in comparison to evidence from literature points towards selective reduction in some of the iron uptake genes (*iroN* and *fyuA*), which were seen in higher distribution in isolates from clinical cases of UTI. This suggests a stronger association of these genes in pathogenesis and makes them candidates for potential pathogenic switch markers which could help in predicting the evolution of the association from commensalic to pathogenic or vice versa.

#### Toxin

Alpha hemolysin virulence gene is necessary for the initial invasion through the epithelial barrier, while cytotoxic necrotizing factor is required for the dissemination and persistence of *E*. *coli* isolates [72]. Our results illustrated that *hlyA* was present in the highest number of isolates (27.5%) followed by *cnf* (22.5%). However, *sat* was only present in less than 8% of the isolates. In a previous study conducted by Watts et al. [70], the occurrence of *hlyA* and *cnf* in ASB strains was 19% and 27%, respectively.

Uropathogenic specific protein (*usp*) is widely disseminated in the UPEC isolates and may act as a bacteriocin that enhances their infectivity in the urinary tract [73]. A previous report has shown that *usp* was associated with *E*. *coli* strains that induce pyelonephritis, prostatitis, and bacteremia [74]. Therefore the occurrence of this virulence gene is often associated with increase in the virulence and fitness of pathogenic strains [75]. Data from the present study revealed that the occurrence of *usp* in ASB strains was 34.3%. In this study, *usp* was highly distributed in phylogroup B2, D, E, and F compared to phylogroups A and B1. This perhaps adds evidence to the argument that *E*. *coli* strains belonging to ExPEC phylogroups are pathogenic even if they were found in ASB cases and *usp* is probably more important to them since it plays a role in virulence and pathogenicity.

As in the case of the adhesion and iron uptake virulence determinants, the toxin encoding genes may logically be selectively lost or acquired depending on the environment. So, in the case of ASB, toxins that aid invasion *(hlyA and cnf)* would be the possible targets to be lost against the ones which could help in survival on mucosal layers (*usp*) as strains shift towards commensalistic existence. Although the natural flow of urine frequently removes some number of bacteria from time to time reducing the need for suppressing the other bacteria, in the battle for space on the mucosal epithelium, the isolates possessing bacteriocin (*usp*) would selectively have a higher survival advantage and so probably retained more in a higher number of isolates. This study does not provide evidence towards these arguments as the toxin genes were detected in comparable distribution among ASB strains and isolates from symptomatic UTI cases.

#### Capsule synthesis

Data generated in this study showed that 38.0% of the *E*. *coli* isolates were positive for *kpsMTII a*nd *ompT* was found in 15% of isolates. The presence of *kpsMTII* was higher in phylogenetic group B2. A previous study reported a similar occurrence of *kpsMTII* (40.5%) in *E*. *coli* isolates from ASB cases [66]. Evidence also suggested that possession of *fimH* (adhesion) and *kpsMTII* (avoidance of phagocytosis) genes simultaneously may enhance the pathogenesis of *E*. *coli* strains.

### Association of virulence genes with phylogroups

In this study, the occurrence of all 12 virulence genes was observed higher among ExPEC than non-ExPEC phylogroups. Among ExPEC, *E*. *coli* from phylogroup B2 showed the occurrence of all virulence genes tested in this study. The genes *iroN*, *cnf*, *hlyA*, *usp*, and *kpsMTII* had a significant association with phylogroup B2.

Although phylogroups A, B1, and C had occurrence for all four virulence functional groups studied, the occurrence were lower in comparison to ExPEC phylogroups. Among adhesion group, *fimH* gene and among iron uptake group *iroN* and *fyuA* genes were observed in higher occurrence in these phylogroups compared to other virulence genes, re-emphasizing the need of these two genes (or the functionality of adhesion and iron acquisition) in colonizing the urinary tract. Non-ExPEC phylogroups have also shown the presence of toxin-associated virulence genes in the lower frequency. All the non-ExPEC phylogroups do not have *chuA* gene, as this gene was a differentiation marker between ExPEC and non-ExPEC phylogroups.

Results obtained in this study revealed that ASB strains from ExPEC phylogroups contained a greater number of virulence genes than the non-ExPEC phylogroups. Findings from this study corresponded with a similar association reported in the literature with UPEC isolates belonging to phylogroups B2 and D from symptomatic UTI cases. Similar to these findings, Dadi et al. [52] and Clara et al. [76] also reported a significant association of phylogroups B2 with *sfa* and *cnf* virulence genes from UPEC isolated from symptomatic UTIs. Although the results of this study showed an association between the presence of virulence genes with ExPEC phylogroups, especially B2, it would be important to take note that there were three strains from the B2 phylogroup that did not have any virulence genes screened in this study except *chuA* as a phylogenetic group marker.

### Association of virulence genes and phylogroups seen in cluster analysis

Data reduction through cluster analysis provided more insights into the data generated in this study. The cluster analysis data, although done statistically, was comparable to genotyping, as the isolates were distributed based on the presence or absence of a genetic element (adhesion iron uptake, toxin, and capsule synthesis genes). This would be the first attempt to cluster the strains separately according to its virulence functional groups, as the existing data of cluster analysis in the literature utilised phylogenetic groups, multilocus sequence typing (MLST), and virulence gene expression data as a whole to cluster the strains. Although the study includes phylogenetic typing, the cluster analysis provides another perspective in our attempt to critically appraise the probability of any isolate being a pathogen. The cluster analysis carried out based on the presence or absence of genes in each functional group resulted in the elucidation of a null cluster for each functional group without any of the genes of that group being present. The respective null clusters for each functional group had 18.1% of isolates in the adhesion group, 52.5% isolates in both iron uptake system and capsule functional groups, and 42.5% isolates in the toxin functional group. The distribution of the phylogroups in the null clusters was not significantly different. As the clusters progressed from null clusters to clusters with permutations and combinations of one of the genes to all of the genes studied, the trend indicated a significant increase in the number of B2 phylogroups isolates being represented in these clusters. This trend was more clearly evident in the toxin functional group and the iron uptake systems functional groups. This would be a trend probably worth looking at considering the fact that these two functional groups of genes are important in the pathogenesis of the UTIs. Near half of these isolates from ASB cases harbours one or more virulent genes from these functional groups and could potentially be a pathogen. These isolates included all the phylogroups and hence genotyping along with detection of some important virulence determinants could lead to the prognostic identification of a pathogenic isolate in ASB cases, which require antibiotic therapy.

### Gene expression analysis

*E*. *coli* isolated from ASB have been widely explored for its prevalence and genotypic characteristics. However, as per our knowledge, this is the first time a study is reporting about the virulence determinants’ profile and their expression among the *E*. *coli* isolated from ASB during pregnancy. The study analysed two ASB isolates, the high (EC095) and low pathogenic potential (EC114) strains, which were observed to be at diverging ends of the spectrum of pathogenicity based on the occurrence of virulence genes studied. The study observed a varying pattern of permutations and combination of the presence of virulence determinants among the *E*. *coli* isolates from ASB.

Data obtained from gene expression studies revealed that EC095 had significantly higher expression of virulence genes in the gene clusters for adhesion, iron uptake, toxin, and capsule synthesis functional groups, in comparison to EC114, at the same time not causing any clinical manifestation in the host. The expression of adhesion virulence gene *fim*, was identified to play an important role in prolonging colonization and survival of ASB isolates in the urinary tract as also was seen in this isolate. Interestingly in this study, EC095 was observed to express *papC* gene, although the expression of this gene is highly reported to be associated with symptomatic UTI.

EC095 also showed significantly higher expression of all iron uptake genes included in this study (*iroN*, *fyuA*, and *chuA*) and related genes of the same gene cluster. The expression of iron uptake-related virulence genes are crucial and may provide a higher survival advantage to this isolate, especially to scavenge the iron, in an iron limiting environment as in the urinary tract. Among the toxin-associated genes, there was also significantly higher expression observed for *cnf* and *hlyA* gene. Similarly to *papC* gene, the presence and expression of these genes were also reported to be associated with symptomatic UTIs. For capsule synthesis virulence functional group, *ompT* and *kpsMTII* genes showed significantly higher expression in this isolate. The expression of these genes were reported to play an important role in biofilm formation, enhancing pathogenicity and high probability of antibiotic resistance. These results indicated extreme polarity of pathogenic potential among ASB isolates as represented by the strains EC095 and EC114.

The study utilised hierarchical cluster analysis based on the presence or absence of genes from an individual functional virulence determinant group to analyse the distribution of phylogroups among the clusters of functional virulence genes. Each cluster analysis produced a null cluster without the presence of the virulence gene and clusters with varying permutations and combinations of virulence genes. The selected EC095 and EC114 were also distributed in extremely diverging clusters, demonstrating a high correlation between cluster analysis and gene expression analysis. This indicates the utility of cluster analysis for the purpose of differentiation of low and high pathogenic potential isolates [Tables 8–11].

The results obtained from this gene expression analysis support the argument that a significant number of *E*. *coli* isolates causing ASB during pregnancy might be with very low pathogenic potential. This further augments the findings from genotypic characterization and hierarchical clustering done in this study. Overall this study identified 48.6% (n = 75) ASB isolates possessed only a combination of any two virulence genes or a lesser number of genes tested in this study. Among these, 68 strains harboured maximum of two virulence genes tested. The majority of these strains are from non-ExPEC phylogroups. The result obtained from gene expression studies justifies the need for treatment decisions for ASB during pregnancy based on isolate characteristics, rather than treating all the cases. This approach, eventually could help to identify the patients with a high risk of getting UTI and thus minimize the consumption of antibiotics.

### Antibiotic resistance

Emergence of antibiotic resistance has been shown to be multifactorial. However, overuse and inappropriate prescription of antibiotics were often reported as main causes for the emergence of MDR. According to European Clinical Disease Prevention and Control, more than 50% *E*. *coli* isolated from clinical samples developed resistance to at least one antimicrobial class and combined resistance to few antimicrobial classes used frequently.

There is contrasting evidence for the effectiveness of treatment of ASB, and so the treatment of ASB is only recommended if it’s associated with a significant health condition such as pregnancy.

Prescription of AMC, SAM, cephalosporins and carbapenem has been widely recommended for UTI treatments. However, the emerging AMR in *E*. *coli* is attributed to ESBL which could destroy various beta-lactam antimicrobials used to treat UTIs such as penicillin, cephalosporins, and carbapenems [19]. In Malaysia, despite having clinical guidelines and antimicrobial stewardship programs, 5% of inappropriate prescribing has been reported in the treatment of ASB [8]. Treating all ASB without determining the virulence potential of the *E*. *coli* isolates and noncompliance to the standard practice and guidelines for ASB might further complicate the infection management.

The present study includes the sensitivity patterns of *E*. *coli* isolates from the ASB cases against 11 different antimicrobial classes as per institutional antibiotic guidelines in the study setting and National Antibiotics Guideline (Malaysia) 2019 [77]. The result from this study was in agreement with previous reports which have shown that resistance to ampicillin was the highest in *E*. *coli* isolated from ASB [78]. The occurrence of resistance to CRO and CTX in this study is comparatively lower than the 100% resistance reported for these antibiotics in Cameroon [79]. The resistance to trimethoprim-sulfamethoxazole in this study is also lower than 60% reported in Somaliland [78]. According to the European Association of Urology guidelines, an agent should be considered for empiric treatment of uropathogenic bacteria if the resistance is less than 20% [80]. In line with this recommendation, the result from this study indicates that the two important antibiotics (AMC and SAM) recommended by the local guideline for ASB in pregnancy should not be considered as an initial treatment regimen in the context of this study. On the other hand, data from this study also revealed that CN and NOR can be safely used for empirical treatment as they showed the lower resistance.

Results in this study also revealed that nearly half of the isolates were MDR and the majority of them are from phylogroup B2. These results were in agreement with previous studies that have demonstrated phylogroup B2 to harbour the majority of MDR strains [81]. Reports of MDR *E*. *coli* among isolates from clinically manifesting UTI cases have shown different distribution such as 13% in Italy [60], 44% in South Korea [72], and 80% in Pakistan [45]. The occurrence of MDR is associated with the indiscriminate use of antibiotics and the dissemination of antibiotic resistance genes in the tested population.

### The trends demonstrated in the association of virulence determinants and phylogroups

The trends indicated a few candidate prognostic virulence markers for the development of clinical symptomatic infections which were *papC/sfa* in the adhesion group, *iroN/fyuA* in the iron uptake group, *hlyA*, and *cnf* in general in the toxin group, and *kpsMTII* in capsule functional group. Although *papC* and *fyuA* genes did not show significantly higher association to pathogenic phylogroups, the reason to include them was their known importance in initiating an immune response during symptomatic UTI. The gene expression results also indicated a significant higher expression of these genes in higher pathogenic potential strain (EC095). These genes in combination with *usp* genes would be a plausible virulence marker combination, which could be explored further to find the trends of switching from commensalistic to pathogenic shift during the association of UPEC with the urinary tract.

The distribution of these genes (*papC/sfa*, *hlyA/cnf*, *iroN/fyuA*, *usp*, *and kpsMTII*) if analysed along with their presence in the pathogenic phylogroups B2, D, E, and F could initiate the development of an algorithm to predict the possibility of development of symptomatic UTI in patients with ASB. This could be explored further in longitudinal studies with follow-up of patients for the development of clinically manifesting UTIs.

The study indicated the utility of cluster analysis as a validity check for phylogenetic typing and an alternate approach to typing. In the absence of phylogenetic data, simple cluster analysis with the information from the frequency of virulence genes would probably provide the required information on trends of association among genes and the strains’ probable virulence nature.

### Limitation

The current study had some study design limitations, as it was an explorative analysis. The study did not utilize a case-control approach, thus, the evidence generated needs further confirmation. The number of isolates among the phylogroups were not comparable and also the frequency of isolation was lower for phylogroups of E, F, and clade I. Even with lower frequency, the trend shown was insightful and indicated the need for extensive exploration with a higher number of isolates. The isolates from this study were not studied for antibiotics resistance genes, thus the correlation between antibiogram phenotype and presence of genes could not be determined. Further study is recommended to determine occurrence of antimicrobial resistance genes to better understand the relationship between phenotypic and genotypic MDR among the *E*. *coli* isolates.

## Conclusion

To our knowledge, this is the first comprehensive work that characterizes the phylogroups, virulence genes profile, and antibiotic susceptibility of *E*. *coli* isolated from pregnant women with ASB in Malaysia. The prevalence of ASB in pregnant women attending study setting were found to exceed the global range of ASB in pregnancy which is 2.5 to 10%. Within phylogroups, the occurrence of ASB strains from phylogroup B2 was higher in comparison with other phylogroups and harboured all of the UPEC associated virulence genes. Overall, *E*. *coli* among phylogroups of A, B1 and B2 exhibits resistance to all antibiotics tested in this study. ASB isolates from this study showed higher resistance to common antibiotics recommended in local guidelines for the treatment of ASB in pregnancy and UTI. Increased MDR among the ASB isolates may serve as a warning for emergence and dissemination of antimicrobial resistance in the local population presumptively due to indiscriminate use of antibiotics. This may warrant a more informed prescription of antibiotics to treat ASB in pregnancy. Hierarchical clustering analysis revealed that *E*. *coli* isolates from ASB harboured virulence genes in different permutations and combination. It was found that ASB isolates from pregnant women represented the two extreme ends of a continuum of the spectrum of its pathogenicity, ranging between absence of virulence genes to presence of most of the virulence genes. It is postulated that *E*. *coli* with null clusters (with no virulence genes) might not be pathogenic thus, might not require treatment even if it is associated with pregnancy. This recommendation also gets considerable validation based on results obtained from the gene expression analysis in this study. The expression analysis of UPEC associated virulence genes, clearly showed that *E*. *coli* from ASB which were clustered into null clusters might be similar to urinary tract colonisers. A significant proportion of the isolates were also closer to the end of the spectrum towards urinary tract colonisers.

Guided with the findings from this study, determination of virulence potential of *E*. *coli* together with evaluation of individual patient characteristics for risk factors of UTI, would enable a better decision making for treatment of ASB cases. The findings of this study could be applied in designing evidence-driven strategies in treating ASB in pregnancy caused by *E*. *coli*. This will facilitate the reduction of the development of resistance among the urinary tract isolates and better utilization of antibiotics.

## Supporting information

S1 File Original data of distribution of phylogroups, virulent determinants and antimicrobial resistance pattern of *E*. *coli* isolates. (XLSX)
